# Prevalence of diabetic retinopathy in Pakistan; A systematic review

**DOI:** 10.12669/pjms.342.13819

**Published:** 2018

**Authors:** Seema N. Mumtaz, Muhammad Faisal Fahim, Muhammad Arslan, Sikander Ali Shaikh, Umer Kazi, Muhammad Saleh Memon

**Affiliations:** 1 Dr. Seema N. Mumtaz, MBBS, M.Phil, MPH, MBA (Health Care).. Isra Ophthalmic Research & Development Center, Karachi, Pakistan; 2 Mr. Muhammad Faisal Fahim, M.Sc.(Statistics). Isra Ophthalmic Research & Development Center, Karachi, Pakistan; 3 Mr. Muhammad Arslan, MCSW. Isra Ophthalmic Research & Development Center, Karachi, Pakistan; 4 Mr. Sikander Ali Shaikh, M.A (Sociology). Community Based Projects; 5 Dr. Umer Kazi, FCPS. Department of Ophthalmology, Al-Ibrahim Eye Hospital, Isra postgraduate Institute of Ophthalmology, Karachi, Pakistan; 6 Dr. Muhammad Saleh Memon, FRCS (Eden), Isra Ophthalmic Research & Development Center, Karachi, Pakistan

**Keywords:** Diabetic Retinopathy, Prevalence, Vision Threatening Diabetic Retinopathy

## Abstract

**Objectives:**

Primary aim was to review the literature on the prevalence of diabetic retinopathy (DR) and Vision threatening diabetic retinopathy (VTDR) in Pakistan.

**Methods:**

A search of the bibliographic databases (Medline, Pub med, and Google scholar) was conducted from 1990 to March 2017. Articles about prevalence of DR and VTDR in Pakistan were retrieved and scrutinized. The studies satisfying the inclusion/exclusion criteria were considered for detail review.

**Results:**

Forty one articles on prevalence of DR were traced out. Exclusion and inclusion criteria were met in 29 studies. In selected studies (29), pooled Prevalence of DR was found to be 28.78% with a variation of 10.6% to 91.3%. Out of 29 studies, DR was classified in 19 studies. Pooled Prevalence of VTDR in these 19 studies was found to be 28.2% (variation of 4% to 46.3%) of patient with retinopathy and 8.6% of all diabetics.

**Conclusion:**

A great variation in the values of DR and VTDR was observed in this study. Researchers suggest a community based study with uniform methodology to find out a comparable value of prevalence of DR and VTDR in all provinces of Pakistan.

## INTRODUCTION

Second national survey on prevalence of blindness in 2004 showed cataract, glaucoma, and corneal disease as common causes of blindness. Posterior segment diseases were responsible for 9.5% as compared to 5.4% in first national survey in 1990. Diabetic retinopathy related blindness (DRB) was not considered in 1990 survey; but in second survey DRB was recorded as < 0.5% amongst the causes of posterior segment disease. Diabetes is increasing and so will be its chronic complications. Studies by King et al Wild et al and Shaw et al have shown that diabetes mellitus is likely to double between 2000 and 2030 mostly in developing countries. In 2010, of an estimated 285 million people worldwide with diabetes, over one-third had signs of DR, and one fourth of these were afflicted with vision-threatening diabetic retinopathy (VTDR), defined as severe non-proliferative DR, proliferative DR (PDR) and diabetic macular edema (DME).

There are sufficient studies from countries with large population like China and India to show the threat from diabetes and its complications^3^,^4^,^5^ and these countries have National plans to prevent the problems of diabetes. Pakistan with more than 200 million (recent census) is expected to have large number of diabetic patients with DR with no plan to combat the consequences. We are lacking in conclusive data highlighting problem of diabetes and DR to generate enough advocacy of the policy makers to plan a “National program” to address diabetes related blindness. In a review article by Hakeem R et al prevalence of diabetes has been quoted as 7.6% to 11%. In a recent press release by Baqai Institute of Diabetology and Endocrinology (BIDE), prevalence of diabetes in Pakistan is 26%. Very little work has been done on DR and VTDR. Values quoted in literature are between 10.6% and 91.34% for DR. Prevalence of VTDR has been quoted between 4%^10^ and 46%. In the present article, Researchers intended to study the screening modalities used in Pakistan, heterogeneity in results and its reasons, flaws in the classifications used for DR and find out pooled statistics for DR and VTDR.

This study was designed to review the articles since 1990 to March 2017 on the prevalence/frequency of DR and VTDR in Pakistan. This data will be helpful for advocacy of the policy makers to consider planning regarding “National program on diabetes related blindness”.

## METHODS

### Appraisal of Study Methodology

This study was approved by “Research Ethical Committee (REC) of Isra Post-graduate Institute of Ophthalmology, Karachi. There were no conflicts among reviewers.

### Research Design and Methods

A systematic literature review was conducted to identify all population-based and hospital-based studies done in Pakistan during 1990 – March 2017.

### Exclusion Criteria

The articles were excluded on basis of nationality (Non Pakistani), duplication, incompleteness, irrelevance and ambiguity of data.

### Inclusion Criteria

Articles and abstracts electronically accessible with DR/VTDR as keyword. All studies having Hospital and/or population-based data for DR/STDR in English language were included.

### Data extraction

Articles were retrieved from Medline, Pub Med and Google scholar by putting search key words, “diabetic retinopathy”, frequency/prevalence and “Pakistan”.

The identified studies were reviewed for authors, study design, duration & place of study, sample size, tools used to detect DR, and scales used to classify DR.

A total of 41 articles were traced in which 35 were full articles and 6 abstracts. Out of these studies, 29 studies fulfilled the inclusion criteria in which 25 were full text articles, 3 abstracts and one thesis. All the studies were published in national journals except one which was published in Turkish journal.

**Fig.1:**
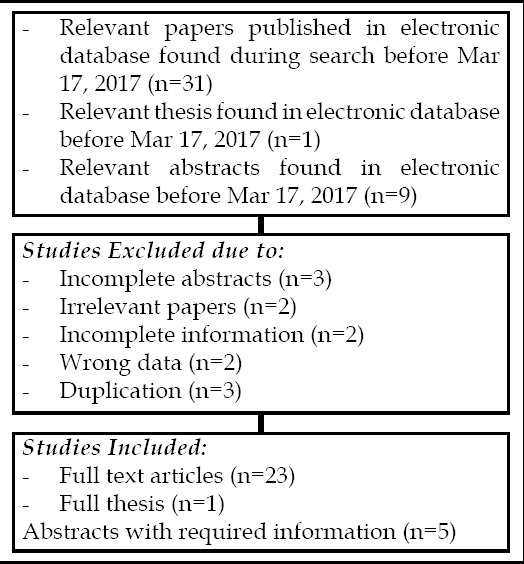
Represents flow chart of selected articles.

All the selected articles were reviewed by following criteria:

### Setting of the retinal screening

Retinal Screening for DR/VTDR was either Hospital based where retinal screening was done in diabetic patients attending a secondary/tertiary centers (Hospital based) for any health problem or community based where screening was done in the community.

### Tools used for retinal screening

The tools used for screening of DR were direct Ophthalmoscopy, indirect ophthalmoscopy, Slit-lamp bio-microscopy with 90D fundus lens in dilated pupil or digital photography with Non-Mydriatic fundus camera (NMFC). In Non-Mydriatic fundus camera, the screening was done through un-dilated pupil taking one 45^0^ retinal image with center to the macula of each eye. Fluorescence Fundus Angiography (FFA) and Optical Coherence Tomography (OCT) were done in selected cases.

### Human resource involved in retinal screening

Screening of retina for retinopathy was mostly done by retina trained ophthalmologist, general ophthalmologist, optometrist, family/general physician and diabetologist.

### Classification or Grading of DR

Classifications used were either “Modified Airlie House / EDTRS classification” or “International Clinical Disease Severity Scale for DR”. Former classification is based on stereo photographs of seven fields and is used as a research tool rather than clinical use. Common classification in use is “International Clinical Disease Severity Scale for DR”. It does not require specialized examinations such as optical coherence tomography or fluorescein angiography. In this classification, five stages are recognized. (Table-A)

**Table-A:** International Clinical Diabetic retinopathy disease severity scale.

*Severity scale*	
Disease serving level	Finding observable upon dilated ophthalmoscopy
No apparent retinoscopy	No abnormalities
Mild NPDR(see glossary)	Microanuerisms only
Moderate NPDR (see glossary)	More than just micro aneurisms but less than severe NPDR
Severe NPDR US definition	Any of the following(4-2-1 rule) and no signs of prolifative retinoscopy Severe intraretinal hemorrhages and microanuerisms in each of four quadrants Definite venous beading in two or more quadrants Moderate IRMA in one or more
International definition	Any of following or no signs of proliferative retinopathy More than 20 intra retinal hemorrhages in each of four quadrants Definite venous beading in two or more quadrantsq Prominent IRMA in one or more quadrants.
PDR	One or both of the following, Neovascularization Vitreous/pre retinal hemorrhage

IRMA= Intraretinal microvascular abnormalities, NPDR= non proliferative diabetic retinopathy,

PDR= proliferative diabetic retinopathy.

NOTE: • Any patient with two or more of the characteristics of severe NPDR is considered to have very severe NPDR. • PDR may be classified as high risk and non high riskWilkinson CP, Ferris FL, Klein RE, et al. proposed international clinical diabetic retinopathy and diabetic macular edema disease severity scales. Ophthalmology. 2003;110:1679.

Diabetic macular edema (DME) is separately described. It is classified as mild, moderate and severe depending on the distance of the exudates and thickening from the center of the fovea. DME can be present alone or in association with any stage of retinopathy. PDR and macular edema are considered “Vision threatening DR (VTDR) whereas mild, moderate and severe non proliferating diabetic retinopathy without macular edema considered is considered as Non-Vision Threatening DR (NVTDR).

### Data Analysis

Statistical Package for Social Sciences Version 20.0 (SPSS Software, Chicago, USA) was used to analyze the data. Frequencies and percentages were calculated for quantitative variable. Pooled Prevalence of DR from 29 studies reported in Table 1. Classification of DR was reported in Table-II. Box plot showed for different Province with respect to prevalence of DR.

**Table-I:** Patients with Prevalence of Diabetes Mellitus & Prevalence of Diabetes Retinopathy (DR) in Pakistan.

Study#	Title	Author	Journal/Year	Type of Study	Sample Size	Tools Used To Detect DR	Frequency of DR (%)	Grading Scale	Types of DR Found
1.	Prevalence of DR in Pakistani Subjects A Pilot Study	Akhtar et al.	JPMA/ 1991	x	3000	Slit Lamp & Top Con Fundus Camera	780 (26%)	x	NPDR = 617 PDR = 163
2.	Presentation of Diabetic Retinopathy	Naeem et al.	JPMI/ 2003	Retrospective cross sectional analysis	100	Bio Microscope Indirect Ophth. & Direct Ophth	38 (38%)	x	NPDR = 28 PDR = 10
3.	Prevalence of Micro Vascular Complications Among Diabetic Patients	Shafiq et al.	PJMS/ 2004	x	573	Direct Ophthalmoscope	102 (55%)	x	X
4.	Prevalence of DR Among Individuals Screened Positive For Diabetes in Five Community Based Eye Camps In Northern Karachi Pakistan	Jamal et al.	J Ayub Med College Abbottabad/ 2006	x	160	90-dioptre Slit lamp Topcon Fundus Camera	17 (10.6%)	x	Mild NPDR =6 (35.3%) Moderate NPDR = 5(29.4%) Severe NPDR =2 (11.8%) PDR =1 (5.9%) Maculopathy 3
5.	Screening for DR: A Comparative Study b/w Hospital & Community Based Screening b/w Paying & Non-paying Patients	Tayyab et al.	J Ayub Med College Abbottabad/ 2007	Comparative study	8227	90 D lens on slit-lamp & indirect Opth.	1834 (22.29%)	x	X
6.	Frequency of Retinopathy In Newly Diagnosed T2DM Patients	Shahid et al.	JPMA/ 2008	Cross sectional	130	x	20 (15%)	x	X
7.	Prevalence of DR & Influence Factors Among Newly Diagnosed Diabetics in Rural & Urban Areas of Pakistan, Data Analysis from the Pakistan National Blindness & Visual Impairment Survey 2003	Aurangzeb et al.	PJMS/ 2008	Survey	660	LogMar, Refraction, Biometry, Un-dilated fundus exam, Slit lamp, Digital photography	101 (15.3%)	x	X
8.	Patterns of Retinopathy Among Diabetic Patients At Tertiary Care Hospital Jamshoro Hyderabad	Ghauri et al.	Medical Channel/ 2010	Descriptive (Case Series) Comparative	100	x	24 (24%)	x	NPDR =18 PDR = 2 Maculopathy 4
9.	Frequency of DR in Patients After 10 Years of Diagnosis of T2DM	Mumtaz et al.	Ayub Med College Abbottabad/ 2010	x	200	x	50 (25%)	x	NPDR = 48 (96%) PDR =2 (4%)
10.	Prevalence of T2DM & DR, The Gadap Study	Pir et al.	JCPSP/ 2010	Descriptive	1677	Used 90 Di-opter lens Indirect Opth.	460 (27.43%)	x	NPDR = 334 (72.61%) PDR = 96 (20.87%) NPDR+CSME = 10 (2.17%) PDR+CSME = 12 (2.61%) Adv. PDR = 8 (1.74%)
11.	Prevalence of Retinopathy & Its Associated Factors in T2DM Patients Visiting Hospitals & Diabetic Clinics in Faisalabad Pakistan	Hassan et al.	Pakistan J. Zool/ 2010	x	500	x	207 (41.4%)	x	X
12.	The Prevalence of DR in Faisalabad, Pakistan A Population Based Study	Fatma et al	TUBİTAK/ 2011	x	1524	Slit Lamp & Stereo Scope	183 (12%)	x	NPDR = 106 (7%) PDR = 77 (5%)
13.	Frequency & Types of DR in Type II Diabetes; A Hospital Based Study	Mehtab et al.	JLUMHS/ 2011	Descriptive case series	244	90 D with the help of slit lamp binocular microscope	100 (40.94%)	ETDRS	Mild NPDR = 61 Moderate - Severe NPDR = 17 PDR = 22
14.	Frequency of DR in Hypertensive Diabetic Patients in Tertiary Care Hospital of Peshawar, Pk.	Shafique et al.	J Ayub Med College Abbottabad/ 2011	Cross sectional	200	Slit Lamp Fundal Fluorescein Angiography	102 (51%)	x	X
15.	Study of DR in Patients Admitted to A Tertiary Care Hospital For Non Opthalmological Reasons	Shafqatullah et al.	Gomal Journal of Medical Sciences/ 2012	Descriptive	462	90D with the help of binocular slit lamp	422 (91.34%)	x	Background DR = 188 Pre-proliferative DR = 172 Proliferative DR = 62
16.	Frequency of DR in a Tertiary Care Hospital Using Digital Retinal Imaging Technology	Aziz et. al	JPMI/ 2012	Descriptive study	2123	Canon CR1 non-mydriatic retinal camera	680 (32.03%)	International Clinical DR Disease Severity Scale	Mild NPDR = (59.3%) Moderate NPDR = (18.7%) Severe NPDR = (14.8%) PDR = (6.4%)
17.	Frequency, Severity & Risk Indicators Of Retinopathy In Patients With Diabetes Screened By Fundus Photographs, A Study From Primary Health Care	Saleh et al.	PJMS/ 2013	Observational	10768	Fundus Camera, Canon CR1	2661 (24.7%) 1650 NPDR 133 PDR CME 878	International Clinical DR Disease Severity Scale	T1DM T2DM Mild NPDR = 59% 45% Moderate NPDR =03% 15% Severe NPDR =0 1% 02% PDR = 0 1% 05% CSME+NPDR = 31% 31% CSME+PDR = 03% 01% Advanced DR = 02% 01%
18.	Risk Factors of Retinopathy in T2DM At A Tertiary Care Hospital, Bahawalpur Pakistan	Sadiq et al.	PJMS/ 2013	Cross sectional descriptive	300	Slit Lamp	74 (23.9%)	x	X
19.	To determine the prevalence of DR in Karachi	Safila et al.	Journal of Scientific and Innovative Research/ 2014	Population based cross sectional survey	150	Bio Microscope & Indirect ophthalmoscope	71 (47.33%)	ETDRS	x
20.	Diabetic Retinopathies & Their Associated Factors, A Study in A Tertiary Care Hospital in Karachi Pk. (Monograph)	Tahir et al.	Thesis, Department Community Med., Univ. of Oslo/ 2014	Retrospective cross sectional analysis	1167	Stereo Scope	853 (73.1%) 761 NPDR 92 PDR	ETDRS & Airline House	Mild NPDR = 395 Moderate NPDR = 321 Severe NPDR = 45 PDR = 92
21.	Sight Threatening DR in T2DM	Memon et al.	Pak Journal Ophthalmology/ 2014	Prospective	200	x	134 (67%)	ETDRS	NPDR = 72 PDR = 62
22.	Frequency And Patterns Of Eye Diseases In Retina Clinic Of A Tertiary Care Hospital In Karachi	Aimal et al.	Pak Journal of Ophthalmology/ 2014	Case study method	3615	20 D and 90 D lenses binocular indirect ophthalmoscope	1440 (39.83%) 840 NPDR 600 PDR+ ADED	x	Bilateral NPDR = 624 (43.3%) NPDR+PDR = 216 (15%) Bilateral PDR = 192 (13.3%) NPDR+ADED = 192 (13.3%) Bilateral ADED = 96 (6.6%) PDR+ADED = 120 (8.3%)
23.	Prevalence of DR Among T2DM Patients in Pakistan – Vision Registry	Mehreen et al.	Pakistan Journal of Ophthalmology/ 2014	Descriptive cross sectional	202	Ophthalmoscope	115 (56.9%)	The Guidelines of Good Epidemiology Practice	Hemorrhages n=70 Cotton Wool Spots n=21 Neo-vascularization n=15 Hard Exudates n=67
24.	Frequency of Diabetic Retinopathy and Microalbuminuria in Newly Diagnosed Type II Diabetes Mellitus patients and their association with each other	Khurram et al.	PJMHS/ 2014	Descriptive case series study	157	Fundoscopy	34 (21.66%)	X	X
25.	Frequency of DR in Karachi, A Hospital Based Study	Saba et al.	Journal of the Dow University of Health Sciences Karachi/ 2015	Cross sectional descriptive	570	Top Con PS-61E Slit lamp Bio Microscope	315 (55.3%)	International Clinical DR Disease Severity Scale	Mild NPDR = 231 Moderate NPDR = 33 Severe NPDR = 11 PDR = 40
26.	Frequency of Diabetic Retinopathy in Type II Diabetics presenting at DHQ Hospital Sahiwal	Khalid et al.	PJMHS/ 2015	Cross sectional study	340	Slit-lamp and 90-D hand held, indirect funduscopy	57 (17%)	ETDRS	NPDR = 50 (87.72%) PDR = 07 (12.28%)
27.	Diabetic retinopathy; Prevalence, among patients attending the free Eye camps for cataract surgery in Southern Punjab, Pakistan	Rasheed et al.	TPMJ/ 2016	Cross sectional study	759	Direct/ indirect ophthalmoscope and slit-lamp, 90-dioptre lens bio-microscope	93 (15%)	ETDRS	NPDR = 87 (93.5%) PDR = 06 (6.5%)
28.	Diabetes Retinopathy Frequency at Level of HbA1c greater than 6.5%	Waseem et al.	Professional Med. J./ 2017	Descriptive case study	130	Funduscopy	31 (23.85%)	International Clinical DR Disease Severity Scale	NPDR = 23 (74.2%) PDR = 08 (25.8%)
29.	Prevalence of Retinopathy Detected by Fundoscopy among Newly Diagnosed Type 2 Diabetic Patients Visiting a Local Hospital in Lahore	Tasnim et al.	PJZ/ 2017	Cross sectional study	200	Fundoscopy	66 (33%)	Retinopathy Disease Severity Scale	PPDR = 14 (7%) PDR = 12 (6%)
30	Total				38,438		11,064 (28.78%)		

**Table-II:** Classification of Diabetic Retinopathy (Total 19 studies).

S#	Study#	Diabetics	DR	%	NPDR=NVTDR	%	PDR+ Macular Edema=VTDR	%
1.	1	3000	753	25.1	617	81.9	163	21.6
2.	2	100	38	38.0	28	73.7	10	26.3
3.	4	160	17	10.6	13	76.5	4	23.5
4.	8	100	24	24.0	18	75.0	6	25.0
5.	9	200	50	25.0	48	96.0	2	4.0
6.	10	1677	460	27.4	334	72.6	126	27.4
7.	12	1524	183	12.0	106	57.9	77	42.1
8.	13	244	100	41.0	78	78.0	22	22.0
9.	15	462	422	91.3	360	85.3	62	14.7
10.	16	2123	680	32.0	631	92.8	49	7.2
11.	17	10768	2661	24.7	1650	62.0	1011	38.0
12.	20	1167	853	73.1	761	89.2	92	10.8
13.	21	200	134	67.0	72	53.7	62	46.3
14.	22	3615	1440	39.8	840	58.3	600	41.7
15.	25	570	315	55.3	275	87.3	40	12.7
16.	26	340	57	16.8	50	87.7	7	12.3
17.	27	759	93	12.3	87	93.5	6	6.5
18.	28	130	31	23.8	23	74.2	8	25.8
19.	29	200	66	33.0	14	21.2	12	18.2
	Total	27339	8377	30.6%	6005	71.7%	2359	28.2%
*DR=Diabetic Retinopathy,						*NPDR= Non-Proliferative Diabetic Retinopathy,		
*NVTDR=Non Vision Threatening Diabetic Retinopathy,						*VTDR=Vision Threatening Diabetic Retinopathy.		

## RESULTS

Total studies on prevalence of DR/VTDR published between 1990 and March 2017, were 41. Studies fulfilling all criteria for review were 29. All these studies were from three provinces, Sindh, Punjab and KPK. No study was reported from Baluchistan or Northern areas. All the studies excluding one were reported in 8 different national journals. One study was published outside Pakistan in Turk J Med Sci.

Majority (24 out of 29) studies were done in hospital setting, four studies (Study # 4, 8, 10 & 17) were community based and only one study (Study # 5) was mixed. The methodology of every study was dissimilar in terms of inclusion/exclusion criteria, tools for DR detection.

### Tools used for screening

Non-Mydriatic fundus camera was used in one study (Study #17) and Mydriatic fundus camera was used in 3 studies (Study # 1, 4, 7). Findings in these 4 studies were confirmed with bio-microscopy. Direct Ophthalmoscopy alone was used in 7 studies (Study # 2, 3, 5, 10, 19, 26, & 27). In reaming 18 studies retinal screening was done by slit lamp bio microscopy using fundus lens.

### Human Resource involved

Personnel involved in screening were ophthalmologist. In one study only (study #17) optometrist used NMFC for screening of DR and referred the DR cases to the retina trained ophthalmologist for grading and intervention.

**Fig.2:**
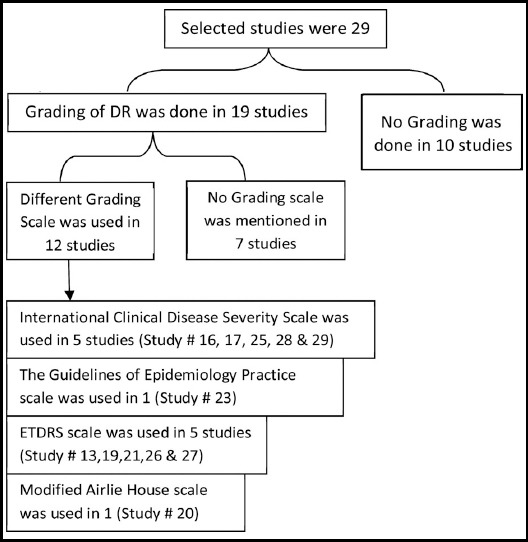
Represents flow chart of DR classification/Grading.

**Fig.3:**
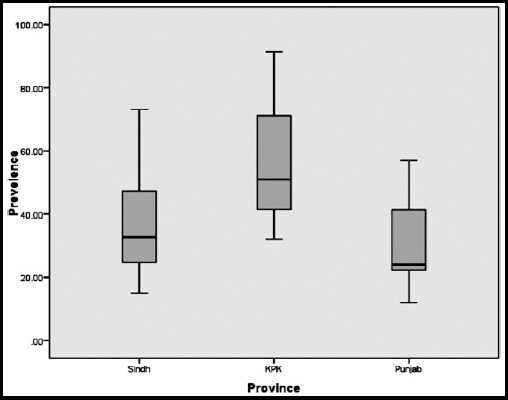
Prevalence of DR according to Provinces.

Macular edema was mentioned only in four studies (Study # 4, 8, 10, and 17). In all of 29 studies a total of 38438 diabetics were screened for diabetic retinopathy (DR). Pooled prevalence of DR was found to be 11064 (28.78%) (With 95% confidence interval [C.I] 29.55 – 47.73) having a huge variation of 91.3% to 10.6%. (Table 1). Amongst 19 studies where DR was classified into VTDR and NVTDR, pooled Prevalence of VTDR was found to be 28.2% (variation 4% to 46.3%) of all DR and 8.6% of all diabetics. (Table 2) When the prevalence of DR was compared between Provinces a large variation in values was found in KPK studies, however in Sindh and Punjab less variation in the data was noted. It was also seen that median line of Punjab was showing less prevalence whereas KPK was showing biggest median in terms of prevalence. (Fig.3)

## DISCUSSION

Pooled prevalence of DR in Pakistan in this study was found to be 28.78% in all diabetics and that of VTDR was 28.2% of all DR and 8.6% of all diabetics (Table 2). DR varies between 10.6% and 91.34%. VTDR varies between 4% and 46%. Huge variations of DR and VTDR in published articles reflect similar values quoted in various national seminars and workshops. This study has explored the reason for inconsistent results. The probable reason of variation in the published articles were e sampling criteria, sample size, duration of study, type of study, methods to detect DR and expertise of the person (ophthalmologist/optometrist). Sample size of at least 12 studies were ≤ than 200. When standard error of proportion was calculated, it was found to be 0.085. This is far too little to prove generalization of results of these review articles for the population. Variation of age group was also not taken into account in many studies. Low frequency can partly be due to failure of detection of DR in early stages especially in cases of diabetic macular edema. Out of 29 studies, macular edema has been mentioned in 4 studies only. Second reason is presence of lens changes masking the fundus. Third reason is the ability of the screener. The effectiveness of different screening modalities has been widely investigated. UK studies show sensitivity levels for the detection of sight-threatening diabetic retinopathy of 41%- 67% for general practitioners, 48%-82% for optometrists, 65% for ophthalmologists, and 27%-67% for Diabetologist and hospital physicians using direct Ophthalmoscopy. The reasons of high prevalence of diabetic retinopathy in some studies could be the area of screening. Screening in a community with lack of awareness, inaccessible and unaffordable eye care service, and lack of knowledge about diabetes and its complications may result in pooling of DR and high frequency. KAP study about diabetics and DR in Gaddap town showed that overall knowledge of diabetes in sample population of (n=527) was 35.23% amongst whom only 7.4 percent respondents considered Diabetic retinopathy as cause of blindness.

With all gaps, the values of DR 28.78% (with 95% confidence interval [C.I] 29.55 – 47.73) and VTDR 8.6% in diabetics are comparable to the values in other developing countries. Prevalence of DR in urban population in Chennai, India was 28.2% (with 95% confidence interval [CI], 27.0–29.3). Liu L et al found the prevalence of DR in China as 23% (95% CI: 17.8%–29.2%) in people with diabetes.

**Note:** Some of the studies included had used the word Frequency along with prevalence as well.

## CONCLUSION

This study provides approximate prevalence estimate of DR and VTDR (PDR, DME) using data from available published studies, mostly hospital based from all over Pakistan. Although published estimates for DR and VTDR varies widely, this study provides an approx. estimates for DR and VTDR high enough to be of significant national public health problem needing urgent attention of policy makers, executives and health care providers.
